# A 3D-printed screw mechanism as an alternative method to prevent wire migration in nonpalpable breast lesion localization

**DOI:** 10.1186/s12893-025-03123-0

**Published:** 2025-08-20

**Authors:** Iksan Tasdelen, Adnan Gundogdu, Kaan Celik, Osman Cem Yilmaz, Levent Celik

**Affiliations:** 1 Fatih Sultan Mehmet Eğitim ve Araştırma Hastanesi, Istanbul, Turkey; 2 Sancaktepe Şehit Prof. Dr. İlhan Varank Eğitim ve Araştırma Hastanesi, Emek Mahallesi, Namık Kemal Cad. No:54, 34785 Sancaktepe, İstanbul, Türkiye; 3 University of Naples Federico II, Naples, Italy; 4 İstanbul Breast Center, Istanbul, Turkey; 5 Radiologica Imaging and Diagnosis Center, Istanbul, Turkey

**Keywords:** Wire migration, Breast surgery, Nonpalpable lesion localization, Screw mechanism, Surgical stabilization

## Abstract

**Background:**

Wire localization for nonpalpable breast lesions and pre-neoadjuvant chemotherapy (NAC) clips can lead to complications including wire migration, pneumothorax, and organ injury. This study evaluates the clinical efficacy and safety of a novel 3D-printed screw mechanism designed to prevent wire migration.

**Methods:**

This retrospective descriptive study included 104 patients with nonpalpable breast lesions and pre-NAC clips who underwent wire localization between March 2023 and August 2024. A screw mechanism was designed and manufactured using 3D printing technology with polylactic acid (PLA) material (4 g per device). The device features a 21.7 mm base diameter and elliptical wire hole (2.5 × 4 mm). Wire stability and complications were assessed through imaging and clinical follow-up.

**Results:**

The median age was 47 years (range: 33–82) and median BMI was 24.7 (17.9–40.8). Lesions were primarily located in the upper outer quadrant (49.9%). Among 68 patients receiving NAC, 44.1% achieved pathological complete response (ypT0N0). The screw mechanism successfully prevented wire migration during patient transport from radiology to operating room in all 104 cases (0% migration rate, 95% CI: 0-3.6%). No complications including pneumothorax, organ injury, or skin reactions were observed. The device was easily removed by simple unscrewing on the operating table before surgical skin preparation. Median device application time was 2–4 h.

**Conclusion:**

The 3D-printed screw mechanism effectively prevents wire migration and demonstrates excellent safety profile. Its simple design and ease of application make it a practical solution for improving patient safety during breast lesion localization procedures.

**Trial registration:**

Not applicable. This retrospective study did not require trial registration.

**Supplementary Information:**

The online version contains supplementary material available at 10.1186/s12893-025-03123-0.

## Introduction

Wire localization is the most commonly used technique in breast surgery for the surgical excision of nonpalpable breast lesions and for guiding surgery after marker clip placement prior to neoadjuvant chemotherapy (NAC), with a majority of units worldwide still relying on this method [1, 2]. As the diagnosis of nonpalpable breast cancers has increased, wire localization guided by mammography and ultrasonography has become increasingly common. This technique accurately localizes tumors, spares uninvolved breast tissue, and enhances the precision of breast-conserving surgery. Although wire localization is generally well-tolerated, it is not without complications. Common issues include bleeding, pain, premature wire removal, and vasovagal reactions. More serious, albeit rarer complications, such as wire fragmentation, migration, pneumothorax, pleural migration, and tumor seeding also pose risks [3, 4].

Wire migration, in particular, presents a significant challenge during patient transport from radiology to the operating room. When wires move from their intended position during this critical period, surgical accuracy is compromised, complicating the procedure. This can lead to difficulties in intraoperative wire tip assessment, which may result in incomplete excisions or the unnecessary removal of healthy tissue [4]. The risk of migration is particularly high during patient positioning, transport, and preparation phases before surgery begins.

Recent meta-analyses have provided valuable data on wire migration rates and alternative localization methods. Shirazi et al. (2023) conducted a comprehensive pooled analysis comparing wire and non-wire localization techniques, demonstrating that non-wire methods had lower positive margin rates compared to traditional wire localization [4]. Additionally, the iBRA-NET study, one of the largest multicenter analyses, documented wire dislodgement in 1.4% of 1,170 wire-guided procedures [5].

Despite the existence of alternative localization techniques such as magnetic seeds (Magseed), radiofrequency identification tags (LOCalizer), radioactive seed localization, and radar reflectors (SAVI Scout), wire localization remains the predominant method. While these wire-free alternatives offer advantages including flexible scheduling, elimination of migration risk, and improved patient comfort, they face significant limitations including requirement for specialized detection equipment, limited availability especially in resource-constrained settings, and learning curves for both radiologists and surgeons [6, 7]. The complications associated with wire localization and the economic burden from re-excisions underscore the need for enhancements to this technique that maintain its accessibility, rather than complete replacement with alternatives that may not be readily available in all clinical settings.

This study introduces an innovative screw mechanism designed to prevent wire migration during breast lesion localization. The mechanism aims to enhance wire stability by preventing both forward and backward movement through a simple mechanical design. Our goal is to assess the efficacy and safety of this newly developed screw mechanism in clinical settings, demonstrating its potential as a practical, cost-effective solution that improves patient safety while maintaining the familiarity and accessibility of traditional wire localization techniques.

## Materials and methods

### Study design

This retrospective descriptive study was conducted between March 2023 and August 2024 to evaluate the effectiveness of a newly developed screw mechanism designed to prevent wire migration during breast lesion localization.

### Patient selection

We assessed 128 consecutive patients, of which 104 patients with nonpalpable breast lesions or clips placed before neoadjuvant chemotherapy (NAC) were included in the study.

The inclusion criteria were as follows:

- Nonpalpable breast lesion requiring localization.
- Age greater than 18 years.
- Scheduled for breast-conserving surgery.

We excluded patients who met any of the following conditions:

-  Multicentric/multifocal disease (*n*=9).
- Diffuse microcalcifications (*n*=6).
- Preference for mastectomy (*n*=5).
- Advanced disease (*n*=2).
- Disease progression during NAC (*n*=2).

For clarity, multifocal disease was defined as having two or more tumor foci within the same quadrant, whereas multicentric disease involved tumor foci in different quadrants or separated by more than 5 cm.

### Design of the screw mechanism

The screw mechanism was developed to prevent wire migration through a simple mechanical design. It consists of two main components:

1. Screw Body: This component includes a cap that provides firm tissue support, thus preventing any forward or backward movement of the wire. The design specifications include a base diameter of 21.7 mm and a total height of 16.8 mm (see Fig. 1). The selected base diameter was intended to distribute pressure over a wide area to minimize tissue compression effectively.

**Fig. 1:**
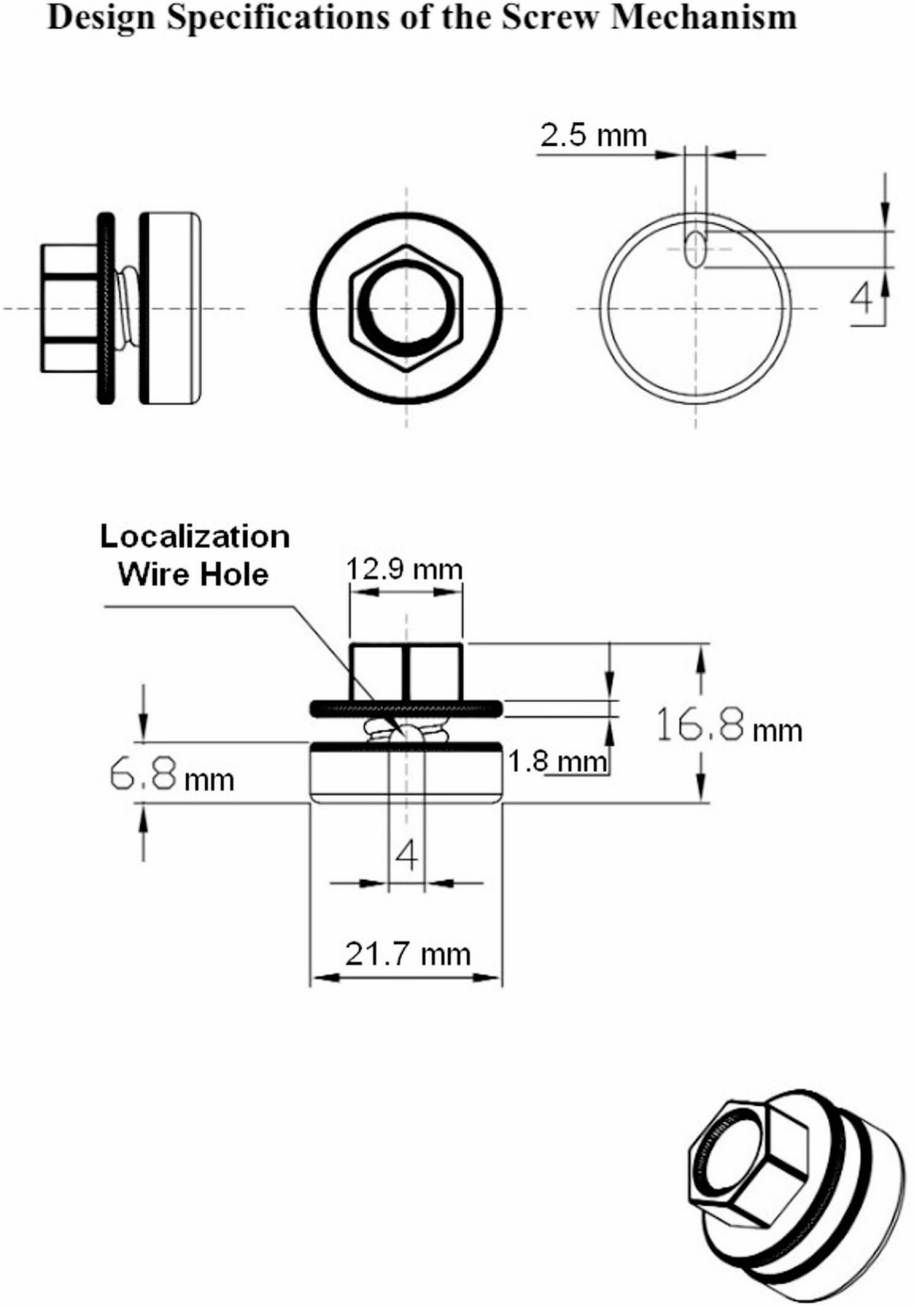
Design specifications of the screw mechanism. Technical specifications of the 3D-printed screw mechanism showing key dimensions: base diameter (21.7 mm), elliptical wire hole (2.5 × 4 mm), total height (16.8 mm), and threading system. The elliptical design prevents wire obstruction during removal
2. Cover Mechanism: This part secures the wire to the screw body via a locking system. The cover is designed with an elliptical hole measuring 2.5 × 4 mm, allowing for unobstructed wire accommodation while preventing slippage. Detailed technical specifications are displayed in Fig. 1. In the cross-sectional view (A-A), the path of the wire as it curves through the top hole and exits through the bottom hole is depicted, illustrating how the design secures the wire in the desired position (see Fig. 2).

**Fig. 2:**
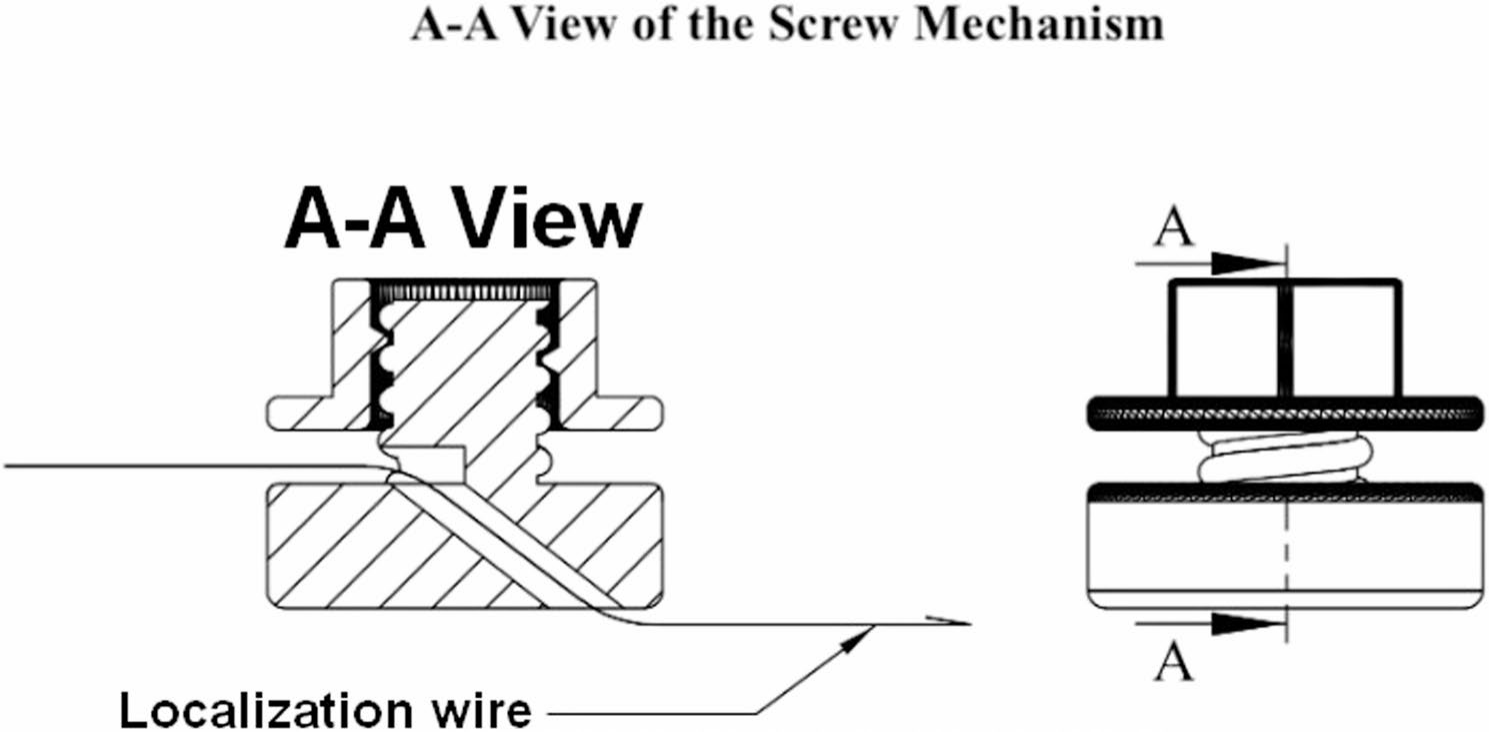
A-A View of the screw mechanism. Cross-sectional view demonstrating the wire path through the mechanism. The wire enters through the top, curves through the elliptical channel, and exits at the bottom, secured by the threaded locking system

### Manufacturing specifications

**Fig. 3:**
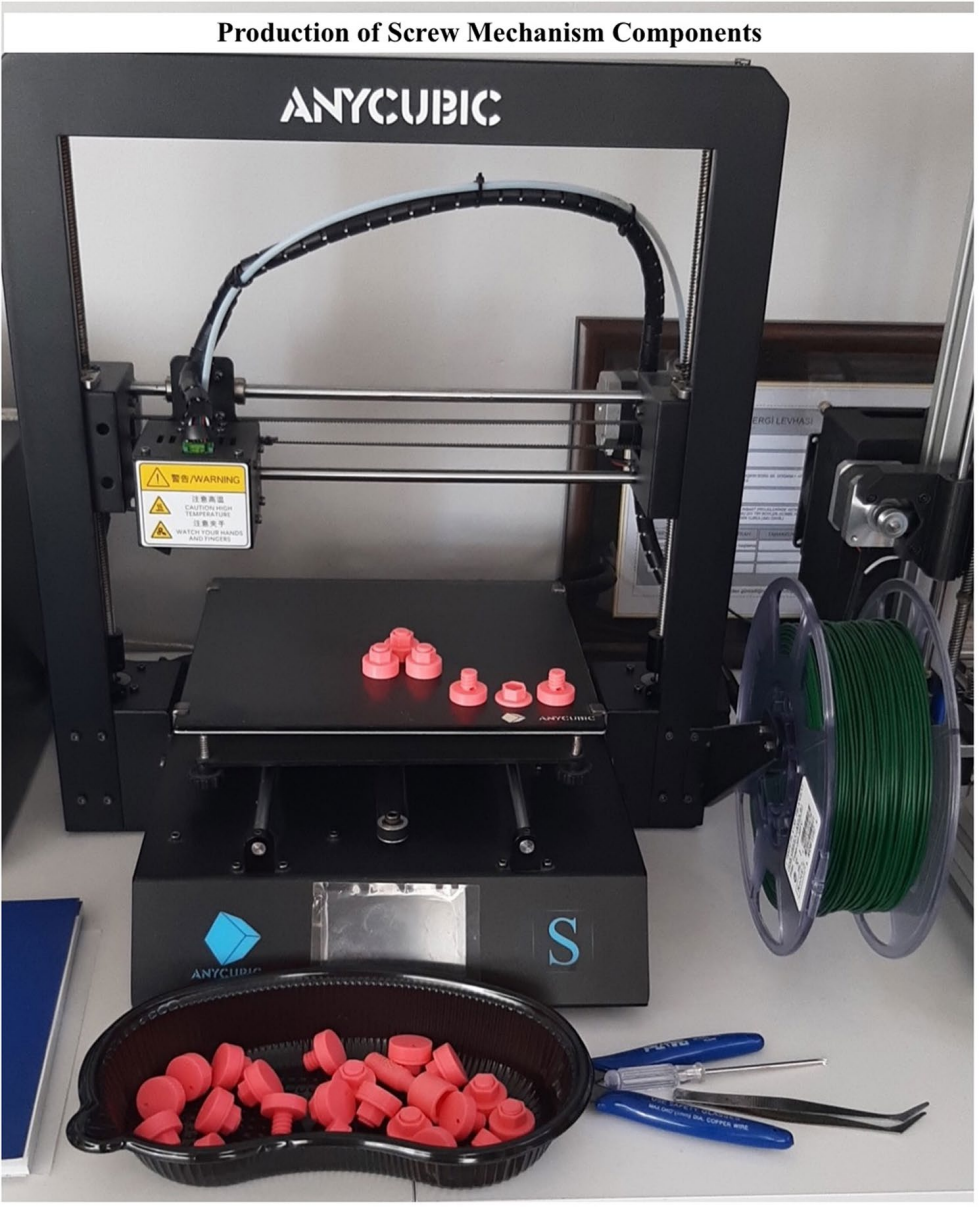
Production of screw mechanism components. 3D printing manufacturing process using FDM technology with PLA material. Shows the ANYCUBIC printer setup and finished devices ready for sterilization and clinical use

The device was manufactured using Fused Deposition Modeling (FDM) 3D printing technology (illustrated in Fig. 3), primarily utilizing polylactic acid (PLA), a biopolymer derived from renewable resources. PLA is a non-toxic thermoplastic known for its environmentally friendly properties, making it suitable for medical applications It is widely used in various fields, including food packaging, healthcare, structural applications, textiles, and cosmetics [8].

### Manufacturing parameters

- Layer resolution: 0.2 mm.
- Infill density: 30%.
- Print temperature: 200 °C (nozzle), 65 °C (bed).
- Print speed: 45 mm/s.
- Material usage: 4 g PLA per device.
- Applied force: approximately 0.04 N (equivalent to 4 g weight).

### Sterilization protocol

All screw components underwent sterilization using ethylene oxide. This sterilization method was selected considering the device’s single-use application during patient transport and its removal before establishment of the surgical sterile field.

### Cost analysis

The production cost per device is approximately $1, broken down as follows:

- PLA material (4 g): $0.30.
- 3D printing time (24 min): $0.40.
- Ethylene oxide sterilization: $0.30.

A utility model patent application has been filed with the Turkish Patent and Trademark Office (Application Number: TR 2023 018924, Filing Date: 27.12.2023).

### Clinical application

**Fig. 4:**
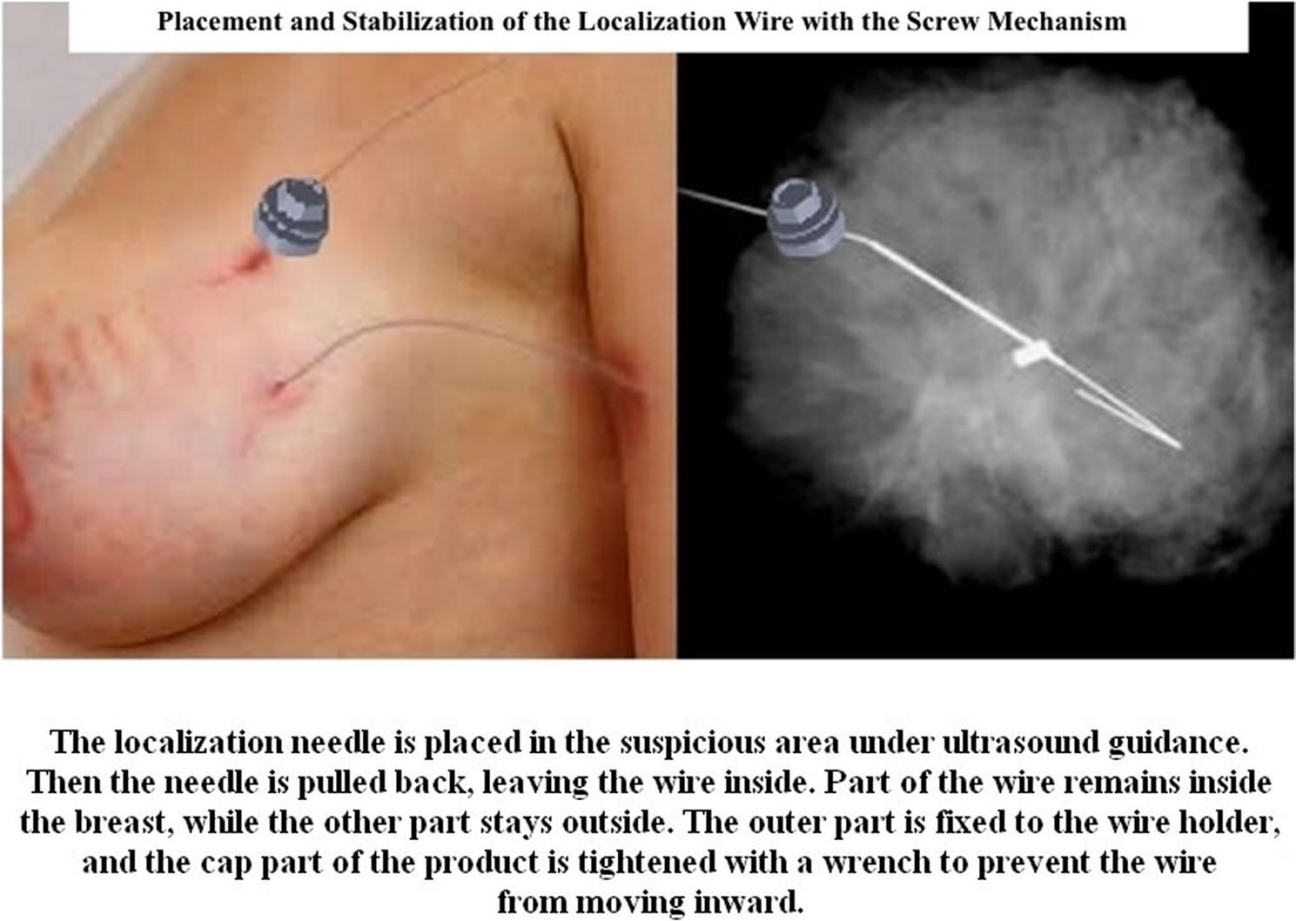
Placement and stabilization of the localization wire with the screw mechanism clinical application demonstrating (left) screw mechanism positioned on patient skin securing the localization wire, and (right) mammographic view showing wire placement within breast tissue

After a thorough evaluation by a multidisciplinary tumor board and preoperative imaging (mammography, ultrasonography, and selective MRI), the localization procedure was performed as (Fig. 4):

1. The surgical approach and incision site were determined by the multidisciplinary tumor board based on tumor location, size, and cosmetic considerations.
2. The localization wire was inserted into the target lesion under ultrasound guidance.
3. The wire was threaded through the screw mechanism’s elliptical hole.
4. The screw was positioned against the skin and tightened to secure the wire.
5. The screw mechanism prevents wire migration by mechanically fixing the wire at the skin entry point during patient transport from radiology to operating room.
6. The device was easily removed by gentle unscrewing on the operating table immediately before surgical skin preparation and draping, without applying force or causing wire displacement.
7. The elliptical hole design (2.5 × 4 mm) allows smooth wire release during device removal without tension or pulling forces on the wire.
8. Maximum application time was limited to 4 h before surgery.
9. Skin assessment was performed before and after device removal to monitor for pressure-related changes.
10. Intraoperative ultrasound was used to ensure complete tumor excision with negative margins.

### Statistical analysis

This was a descriptive study without a control group. Data were analyzed using R version 4.3.0. Descriptive statistics were presented as median (range) for continuous variables and n (%) for categorical variables. The 95% confidence interval for the zero wire migration rate (0/104) was calculated using the Wilson score method with the ‘binom’ package in R, yielding 95% CI: 0-3.6%. Due to the descriptive nature of the study and the absence of complications, comparative statistical tests and p-value calculations were not applicable.

### Ethical approval

 The study protocol was approved by the Ethics Committee of Sancaktepe Şehit Prof. Dr. İlhan Varank Training and Research Hospital (Approval Number: 2024/294, Date: 18.09.2024).

## Results

### Patient characteristics

 A total of 104 patients underwent wire localization using the screw mechanism. The median age was 47 years (range: 33-82 years), and the median body mass index (BMI) was 24.7 kg/m² (range: 17.9-40.8). Lesion distribution by quadrant was as follows: upper outer (49.9%), upper inner (15.4%), central (13.5%), lower outer (13.5%), and lower inner (7.7%). Multisentric/multifocality disease was observed in 14 patients (13.5%). Preoperative pathology revealed invasive ductal carcinoma (IDC) in 56 patients (53.8%), benign findings in 20 patients (19.2%), ductal carcinoma in situ (DCIS) in 14 patients (13.6%), invasive lobular carcinoma (ILC) in 6 patients (5.8%), and mixed carcinoma (ILC and IDC) in 4 patients (3.8%) (Table 1).

**Table 1:** Demographics and clinical characteristics of the patients

Variable		All Patients (*n* = 104)
Age (median, min-max)		47 (33–82)
BMI (median, min-max)		24.7 (17.9–40.8)
Side	Left Right	70 (67.3) 34 (32.7)
Lesion quadrant	Upper Outer Quadrant (UOQ) Upper Inner Quadrant (UIQ) Central Lower Outer Quadrant (LOQ) Lower Inner Quadrant (LIQ)	52 (49.9) 16 (15.4) 14 (13.5) 14 (13.5) 8 (7.7)
Multisentric/Multifocality	Absent Present	90 (86.5) 14 (13.5)
Axillary marking	Absent Present	100 (96.2) 4 (3.8)
Preoperative pathology	IDC ILC Mixed (ILC & IDC) Tubular Carcinoma DCIS LCIS Benign	56 (53.8) 6 (5.8) 4 (3.8) 2 (1.9) 14 (13.6) 2 (1.9) 20 (19.2)

*BMI* Body Mass Index, *IDC* Invasive Ductal Carcinoma, *ILC* Invasive Lobular Carcinoma, *DCIS* Ductal Carcinoma In Situ, *LCIS* Lobular Carcinoma In Situ

### Neoadjuvant chemotherapy subgroup

 Among the 68 patients who received neoadjuvant chemotherapy, the median age was 48 years (range: 33-82) and the median BMI was 25.5 kg/m² (range: 17.9-40.8). Clinical staging showed T1N1 as the most common presentation (29.4%), followed by T2N1 (23.5%) and T2N0 (20.6%). Most lesions were located in the upper outer quadrant (47.1%), and 20.6% had multifocal disease. The median pre-NAC lesion size was 1.9 cm (range: 0.5-4.6 cm). Following NAC, a pathological complete response (pCR) was achieved in 30 patients (44.1%), while 38 patients (55.9%) had residual disease. The predominant molecular subtype was luminal B (44.1%), followed by luminal A (26.5%), HER2-positive (17.6%), and triple-negative (11.8%) (Table 2).

**Table 2:** Demographics and clinical characteristics of the patients who received neoadjuvant chemotherapy

Variable		Patients (*n* = 68)
Age (median, min-max)		48 (33–82)
BMI (median, min-max)		25.5 (17.9–40.8)
Lesion quadrant	Upper Outer Quadrant (UOQ) Upper Inner Quadrant (UIQ) Central Lower Outer Quadrant (LOQ) Lower Inner Quadrant (LIQ)	32 (47.1) 12 (17.6) 10 (14.7) 10 (14.7) 4 (5.9)
Lesion size (cm) (median, min-max)		1.9 (0.5–4.6)
Multicentric/Multifocality	Absent Present	54 (79.4) 14 (20.6)
cTN stage	T1N0 T1N1 T2N0 T2N1 T2-3N2-3	10 (14.7) 20 (29.4) 14 (20.6) 16 (23.5) 8 (11.8)
Pathological response	Complete response (pCR) Residual disease	30 (44.1) 38 (55.9)
Molecular subtype	Luminal A Luminal B HER2 - positive Triple-negative	18 (26.5) 30 (44.1) 12 (17.6) 8 (11.8)

*BMI* body mass index, *cm *centimeter, *cTN *clinical tumor-node stage, *NAC* neoadjuvant chemotherapy, *pCR *pathologic complete response, *HER2 *human epidermal growth factor receptor 2

### Wire migration

No wire migration occurred in any of the 104 patients (0%, 95% CI: 0-3.6%). Intraoperative ultrasound confirmed wire stability and accurate positioning in all cases.

### Surgical margins

All 104 patients (100%) achieved negative surgical margins on final pathology. Intraoperative frozen section analysis and ultrasound guidance were routinely employed to ensure complete excision.

### Device removal

All 104 devices were successfully removed by gentle unscrewing without applying force. No wire displacement occurred during device removal. The elliptical hole design allows smooth wire release without tension, eliminating the risk of wire migration during removal. Intraoperative ultrasound confirmed accurate wire positioning in all cases following device removal.

### Complications

No procedure-related complications were observed, including:

- Pneumothorax (0/104)
- Organ injury (0/104)
- Bleeding requiring intervention (0/104)
- Infection (0/104)

### Patient tolerance

All patients reported no discomfort related to the screw mechanism. Skin assessments revealed no pressure-related changes, erythema, or induration at the device application site.

## Discussion

This study assesses the effectiveness and safety of an innovative screw mechanism designed to prevent wire migration during patient transport for localization of non-palpable lesions and post-NAC clips in breast surgery. The primary objective of the screw mechanism is to enhance patient safety during the critical transport period from radiology to the operating room by effectively ensuring wire stability. Specifically, by preventing wire migration during transport and positioning, accurate localization of lesions is maintained, thereby increasing the success of subsequent surgical procedures.

In this investigation, no complications were noted following the utilization of the screw mechanism. This result underscores the likelihood that the mechanism could be effective in avoiding severe complications such as pneumothorax, organ injuries, and wire migration cited in existing literature [9, 10]. Additionally, it is believed to improve surgical outcomes by lessening the necessity for re-excision owing to positive surgical margins arising from wire migration. Literature indicates that wire migration in conventional methods may extend operation times and necessitate further interventions for patients [4, 11]. Although tested on a limited number of patients in this study, it is quite plausible that the mechanism employed could instill greater confidence in surgeons and radiologists by diminishing the risk of complications.

While other localization techniques such as magnetic localization (Magseed), Savi-scout reflectors, and radio frequency identification (RFID) tags are efficient in locating breast masses, they may encounter accessibility challenges due to higher costs and restricted availability [12–15]. The screw mechanism introduced in our research presents a more affordable and easily applicable alternative while ensuring wire stability. This method, which can be seamlessly implemented with existing instruments, enhances patient safety and streamlines the surgical process. The screw mechanism distinguishes itself as an appropriate option for a broader patient population and demographics owing to its cost-effectiveness and easy accessibility. Hook-wire localization techniques, widely used internationally, rely on tissue anchoring but can still experience displacement complications [7, 16]. Our external screw mechanism provides additional security during patient transport, complementing the wire’s tissue positioning and demonstrating potential as a practical solution in breast surgery.

The screw mechanism devised in this study was designed with an elliptical hole structure to prevent the wire from becoming stuck during exit. If the hole were entirely circular, the wire’s stability could be adversely impacted. The slight tilt in the design enhances safety while allowing for a more controlled wire exit. In pulmonary nodule marking systems, specialized stabilization methods such as pure gold cylinders with knurled surfaces are employed to avoid wire migration [17]. Additionally, cement-supported K-wire frameworks have been highlighted as a secure method to deter wire migration and displacement in orthopedics, particularly in distal radius fractures [18]. In neurosurgery, various stabilization mechanisms are applied to secure deep brain stimulation electrodes [19]. This system, relevant to breast surgery, presents an efficient and innovative solution to wire slippage.

While our results are encouraging, direct comparison with standard wire localization was not performed due to the descriptive nature of this study. The absence of a control group limits our ability to definitively claim superiority over existing techniques. Furthermore, we did not collect patient-reported outcome measures or long-term follow-up data, which are essential for comprehensive evaluation of any localization technique. The immediate postoperative outcomes and 4-hour application period provide only a limited assessment window for device safety and efficacy.

This study has several important limitations that must be acknowledged. The descriptive design without a control group precludes direct statistical comparison with standard wire localization techniques and limits our ability to definitively claim superiority over existing techniques. Additionally, the absence of long-term follow-up data and patient-reported outcome measures limits our assessment to immediate perioperative results within a 4-hour application window. Although the complete absence of complications is encouraging, it prevented meaningful statistical analysis of potential risk factors or predictive variables. Furthermore, conducting this study at a single specialized center may limit the generalizability of our findings to institutions with varying levels of expertise and resources. The device also awaits regulatory approval for routine clinical implementation. These limitations underscore the need for multicenter randomized controlled trials with extended follow-up periods to validate our preliminary findings.

In conclusion, this study sought to evaluate the efficacy of the newly developed screw mechanism in preventing wire migration and promoting patient safety. Its straightforward design, affordability, and practical applicability position this mechanism as a potentially valuable alternative for broader use in clinical environments. The results indicate that this mechanism shows promise as a practical solution for wire stabilization. However, future research should more comprehensively explore the applicability and effectiveness of this mechanism across diverse patient populations through controlled comparative studies.

## Supplementary Information

Supplementary Material 1.

Supplementary Material 2.

Supplementary Material 3.

Supplementary Material 4.

## Data Availability

The manuscript presents the data. Upon request, the corresponding author can provide the datasets analyzed within this study.
