# Supplementary material for: A 3D-printed screw mechanism as an alternative method to prevent wire migration in nonpalpable breast lesion localization
Source: BMC Surg. 2025 Aug 20;25:377. doi: 10.1186/s12893-025-03123-0 (PMC12366026; doi:10.1186/s12893-025-03123-0)
Supplement: Supplementary file 1 — Supplementary Material 1. [file 12893_2025_3123_MOESM1_ESM.pdf]

T.C.
İSTANBUL VALİLİĞİ
İl Sağlık Müdürlüğü
Şehit Prof. Dr. İlhan Varank Eğitim ve Araştırma Hastanesi

Sayı : E-46059653-050.99-254455650 24.09.2024
Konu : Uzm. Dr. Adnan GÜNDOĞDU Bilimsel Araştırmalar Etik Kurul Toplantısı Hk.

Sayın Uzm. Dr. Adnan GÜNDOĞDU

Hastanemiz Sancaktepe Şehit Prof. Dr. İlhan Varank Eğitim ve Araştırma Hastanesinde 18.09.2024 tarihinde yapılan Bilimsel Araştırmalar Etik Kurul toplantısında Ek 1 de yer alan bir adet dosyanız incelenmiş olup "Nonpalpabl Meme Lezyonlarının Lokalizasyonu İçin Kullanılan Telin Stabilitesini Sağlamak Amacıyla Geliştirilen Yenilikçi Vida Mekanizması " isimli çalışmanızın gerçekleştirilmesinde etik açıdan bir sakınca olmadığına toplantıya katılan etik kurul üye tam sayısının salt çoğunluğu ile karar verilmiştir.

2024/294 sayı numaralı Kurul Karar Formu Ek-2 de yer almaktadır.

Gereğini rica ederim.

Doç. Dr. Elif Torun PARMAKSIZ
Etik Kurul Başkan Yardımcısı

Ek:
1 - UZM. DR. ADNAN GÜNDOĞDU.pdf
2 - UZM. DR. ADNAN GÜNDOĞDU BİLİMSEL ARAŞTIRMALAR ETİK KURUL KARAR FORMU.pdf

Bu belge, güvenli elektronik imza ile imzalanmıştır.
Belge doğrulama kodu: 0328098F-68E4-45D3-A80C-CC58B4D501BC Belge doğrulama adresi: https://www.turkiye.gov.tr/saglik-bakanligi-ebys

Emek Mah.Namık Kemal Cad.No:54 Sancaktepe İSTANBUL
34000
Telefon No: 02166063300
e-Posta: İnternet Adresi: https://www.saglik.gov.tr/
Kep Adresi:

Bilgi için: Fatma ATA
Asistan
Telefon No: 21660633001311
