# Supplementary figures and images for: A 3D-printed screw mechanism as an alternative method to prevent wire migration in nonpalpable breast lesion localization
Source: BMC Surg. 2025 Aug 20;25:377. doi: 10.1186/s12893-025-03123-0 (PMC12366026; doi:10.1186/s12893-025-03123-0)

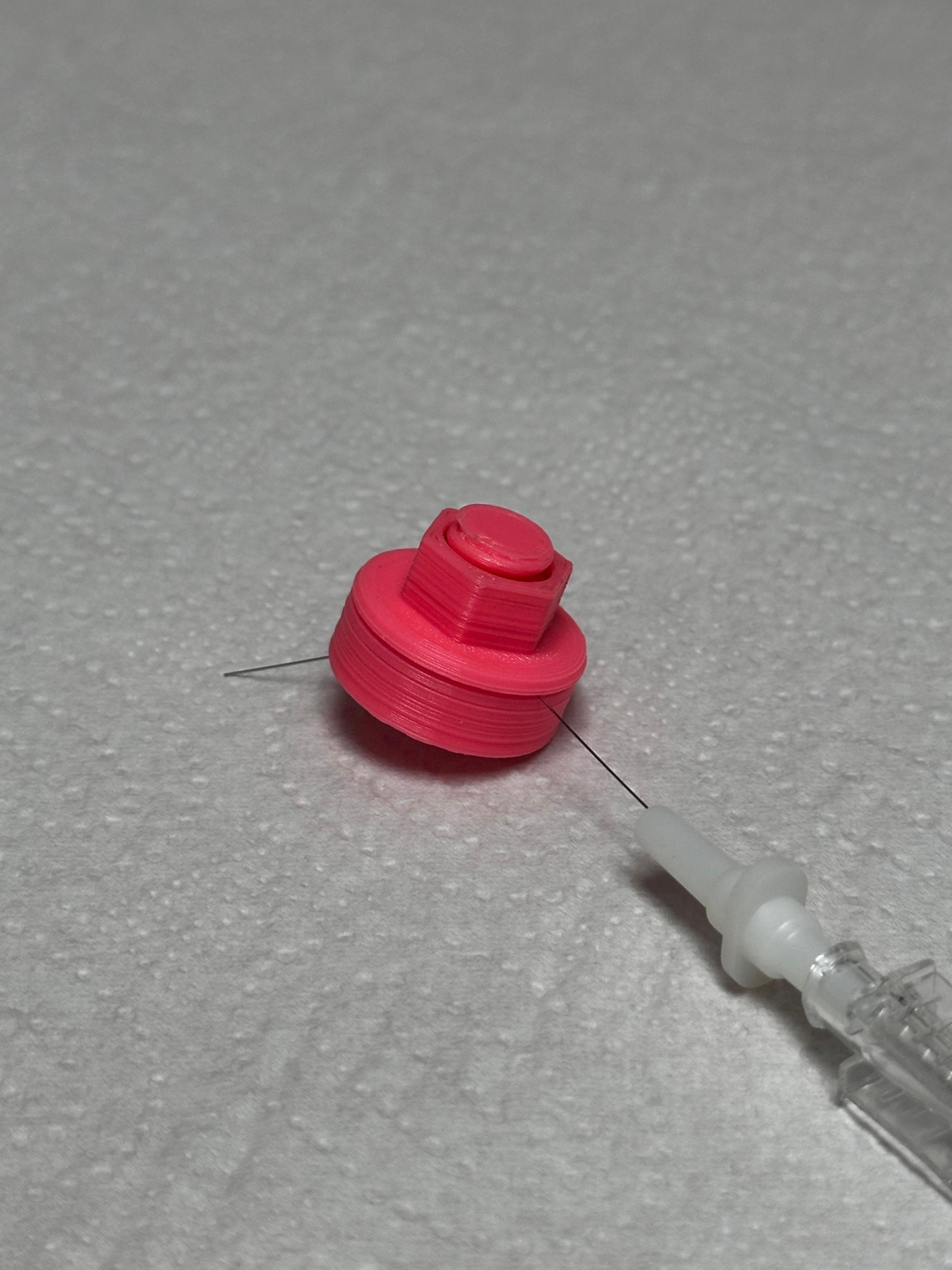

Supplement: Supplementary file 2 — Supplementary Material 2. [file 12893_2025_3123_MOESM2_ESM.jpeg]

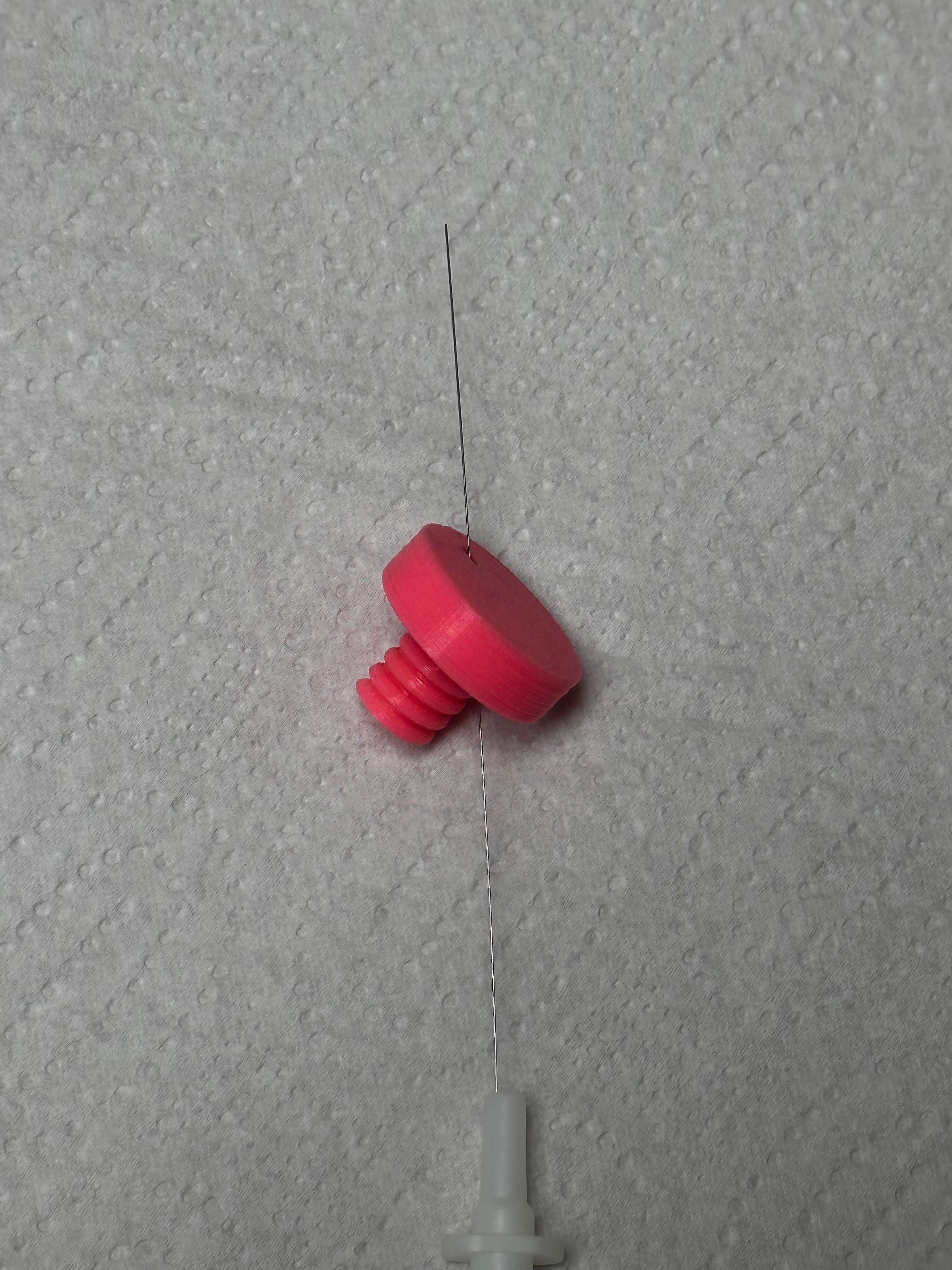

Supplement: Supplementary file 3 — Supplementary Material 3. [file 12893_2025_3123_MOESM3_ESM.jpeg]
