# Supplementary material for: A 3D-printed screw mechanism as an alternative method to prevent wire migration in nonpalpable breast lesion localization
Source: BMC Surg. 2025 Aug 20;25:377. doi: 10.1186/s12893-025-03123-0 (PMC12366026; doi:10.1186/s12893-025-03123-0)
Supplement: Supplementary file 4 — Supplementary Material 4. [file 12893_2025_3123_MOESM4_ESM.pdf]

T.C.
TÜRK PATENT VE MARKA KURUMU
Patent Dairesi Başkanlığı

Sayı : E-39616753-110-250402727 25.06.2025
Konu : Pt/fm Belge Kararı -2023/018924 Numaralı Başvuru

ZELAL ŞENGÜLER (NEOMARK PATENT OFİSİ DANIŞMANLIK LTD. ŞTİ.)

İlgi : 10.06.2025 tarihli faydalı model belgesi düzenlenmesi talebiniz.

İlgide kayıtlı talebiniz üzerine düzenlenen TR 2023 018924 Y sayılı faydalı model belgesi ilişikte gönderilmektedir.

6769 sayılı Sınai Mülkiyet Kanunu ve anılan Kanunun Uygulanmasına Dair Yönetmeliğin ilgili maddeleri kapsamında faydalı model hakkına ilişkin açıklayıcı bilgiler aşağıda verilmektedir.

Faydalı modelin verilmiş olması, geçerliliği ve yararlılığı konusunda Kurumumuz tarafından garanti verildiği şeklinde yorumlanamaz ve Kurumun sorumluluğunu da doğurmaz.

Faydalı model verilmesinden sonra 6769 sayılı Kanunun 99 uncu maddesinde belirtilen itiraz usulü işletilemez, sadece mahkemeden hükümsüzlük talep edilebilir.

Bir faydalı modelin korunması için gerekli olan yıllık ücretler üçüncü yıldan başlamak üzere her yıl (faydalı modelin koruma süresi boyunca) vadesinde ödenir. Vade tarihi, başvuru tarihine tekabül eden ay ve gündür. Yıllık ücretler, vadesinde ödenmediği takdirde ek ücretle birlikte vadeyi takip eden altı ay içinde de ödenebilir. Yıllık ücretlerin bu süre içinde de ödenmemesi halinde faydalı model hakkı, bu ücretin son ödeme tarihi itibariyle sona erer. Ancak, faydalı model hakkının sona erdiğine ilişkin bildirim tarihinden itibaren iki ay içinde telafi ücretinin ödenmesi halinde faydalı model hakkı, ücretin ödendiği tarih itibariyle yeniden geçerlilik kazanır ve Bültende yayımlanır. Söz konusu telafi ücretinin ödenmemesi durumunda 6769 sayılı Kanunun 145 inci maddesinin birinci fıkrası ve 101 inci maddesinin dördüncü fıkrası hükümleri uyarınca hakların yeniden tesisi için talepte bulunulabilir.

Faydalı model sahibi veya yetkili kıldığı kişi, faydalı modelle korunan buluşu kullanmak zorundadır. Kullanma zorunluluğu, faydalı modelin verildiğine ilişkin ilanın ilgili bültende yayınlandığı tarihten itibaren üç yıllık veya faydalı model başvurusu tarihinden itibaren dört yıllık sürelerden hangisi daha geç sona eriyorsa, bu süre içinde gerçekleştirilir. 6769 sayılı Kanunun Uygulanmasına Dair Yönetmeliğin 123 üncü maddesinin birinci fıkrası ve 117 nci maddesinin sekizinci fıkrası hükümleri uyarınca faydalı modelin kullanıldığına ya da kullanılamadığına ilişkin beyanın anılan süre içinde Kuruma sunulması halinde bu durum sicile kaydedilerek Bültende yayımlanır. Bu süre içinde kullanıldığına dair bildirim yapılmayan faydalı modeller Bültende yayımlanır.

Türk Patent ve Marka Kurumunun sunduğu hizmetlere ilişkin ücretlerde, ödemenin yapıldığı tarih itibariyle yürürlükte olan Ücret Tebliğini dikkate almanız gerekmektedir.

Saygılarımla.

Bu belge, güvenli elektronik imza ile imzalanmıştır.
Belge Doğrulama Adresi : https://belgedogrulama.turkpatent.gov.tr/bg.aspx?Id=9CD69299-C49B-4744-9605-22ABF61D89F9
Gazi Mahallesi Hipodrom Caddesi No:13 (06560)
Yenimahalle / ANKARA
Telefon No: (0312) 303 1 303 Faks No : (0312) 303 11 73
İnternet Adresi www.turkpatent.gov.tr
Kep Adresi : tpe@hs01.kep.tr

Bilgi İçin: Tuğçe TUNCAR
Unvan: Programcı
Telefon No:
Eposta: tugce.tuncar@turkpaten.gov.tr

Tuğçe TUNCAR
Başkan a.
Programcı

EK :
1- Ek Listesi (1 Sayfa)

Bu belge, güvenli elektronik imza ile imzalanmıştır.
Belge Doğrulama Adresi : https://belgedogrulama.turkpatent.gov.tr/bg.aspx?Id=9CD69299-C49B-4744-9605-22ABF61D89F9
Gazi Mahallesi Hipodrom Caddesi No:13 (06560)
Yenimahalle / ANKARA
Telefon No: (0312) 303 1 303 Faks No : (0312) 303 11 73
İnternet Adresi www.turkpatent.gov.tr
Kep Adresi : tpe@hs01.kep.tr

Bilgi İçin: Tuğçe TUNCAR
Unvan: Programcı
Telefon No:
Eposta: tugce.tuncar@turkpaten.gov.tr

Ek-1

EK LİSTESİ

1- PT_2023-018924_2025-O-437130_Ek1.pdf

2- PT_2023-018924_2025-O-437130_Ek2.pdf

Bu belge, güvenli elektronik imza ile imzalanmıştır.
Belge Doğrulama Adresi : https://belgedogrulama.turkpatent.gov.tr/bg.aspx?Id=9CD69299-C49B-4744-9605-22ABF61D89F9
Gazi Mahallesi Hipodrom Caddesi No:13 (06560)
Yenimahalle / ANKARA
Telefon No: (0312) 303 1 303 Faks No : (0312) 303 11 73
İnternet Adresi www.turkpatent.gov.tr
Kep Adresi : tpe@hs01.kep.tr

Bilgi İçin: Tuğçe TUNCAR
Unvan: Programcı
Telefon No:
Eposta: tugce.tuncar@turkpaten.gov.tr

(19) TÜRK PATENT — TÜRK PATENT VE MARKA KURUMU

(10) TR 2023 018924 Y

FAYDALI MODEL BELGESİ

(21) Başvuru Numarası
2023/018924
(22) Başvuru Tarihi
2023/12/27
(43) Başvuru Yayın Tarihi
2024/01/22
(11) Başvuru Yayın No.
TR 2023 018924 U5
(45) Faydalı Model Belgesinin Veriliş Tarihi
2025/06/23

(51) Buluşun tasnif sınıfları
A61B 17/34
A61B 19/00

(30) Rüçhan Bilgileri (32) (33) (31)

(74) Vekil
ZELAL ŞENGÜLER (NEOMARK PATENT OFİSİ DANIŞMANLIK LTD. ŞTİ.)
YUKARI DUDULLU MAH. BAYRAK CAD. NO:30 K:3 D:22 BİLİM TOWER ÜMRANİYE/İSTANBUL

(71) Faydalı Model Sahipleri
OSMAN CEM YILMAZ
SUADİYE MAH. SELİM RAGIP EMEÇ SK. KAFAOĞLU APT NO: 13 İÇ KAPI NO: 7 KADIKÖY / İSTANBUL Kadıköy İstanbul TÜRKİYE
BURAK GÜMÜŞ
KAZIM KARABEKİR MAH. 311. SK. IŞIK APT. NO: 48 İÇ KAPI NO: 7 BAĞCILAR / İSTANBUL Bağcılar İstanbul TÜRKİYE
MERVE DUMAN KAVUK
ŞİRİNEVLER MAH. MAHMUTBEY 19. SK. NO: 13 İÇ KAPI NO: 11 BAHÇELİEVLER / İSTANBUL Bahçelievler İstanbul TÜRKİYE
MEHMET SÜHA YÜKSEL
İÇERENKÖY MAH. MİLAT SK. ALTIN APT NO: 15 İÇ KAPI NO: 8 ATAŞEHİR / İSTANBUL Ataşehir İstanbul TÜRKİYE

(72) Buluşu Yapanlar
OSMAN CEM YILMAZ
SUADİYE MAH. SELİM RAGIP EMEÇ SK. KAFAOĞLU APT NO: 13 İÇ KAPI NO: 7 KADIKÖY / İSTANBUL Kadıköy İstanbul TÜRKİYE
MEHMET SÜHA YÜKSEL
İÇERENKÖY MAH. MİLAT SK. ALTIN APT NO: 15 İÇ KAPI NO: 8 ATAŞEHİR / İSTANBUL Ataşehir İstanbul TÜRKİYE
MERVE DUMAN KAVUK
ŞİRİNEVLER MAH. MAHMUTBEY 19. SK. NO: 13 İÇ KAPI NO: 11 BAHÇELİEVLER / İSTANBUL Bahçelievler İstanbul TÜRKİYE
BURAK GÜMÜŞ
KAZIM KARABEKİR MAH. 311. SK. IŞIK APT. NO: 48 İÇ KAPI NO: 7 BAĞCILAR / İSTANBUL Bağcılar İstanbul TÜRKİYE

(54) Buluş Başlığı

MEME KANSERİ AMELİYATLARINDA TELLE İŞARETLEMEDE KOMPLİKASYONLARIN ÖNLENMESİNDE KULLANILAN APARAT

(57) Özet

Buluş, tel tutucu (3), tel tutucu (3) üzerinde vidalı kapak (4) ve işaretleme teli yuvası (2) ile oluşturulan meme kanseri ameliyatlarında telle işaretlemede komplikasyonların önlenmesinde kullanılan aparat ile ilgilidir.

TÜRK PATENT
TÜRK PATENT VE MARKA KURUMU

FAYDALI MODEL BELGESİ

No: TR 2023 018924 Y

Buluş Başlığı

MEME KANSERİ AMELİYATLARINDA TELLE İŞARETLEMEDE KOMPLİKASYONLARIN ÖNLENMESİNDE KULLANILAN APARAT

Faydalı Model Sahibi

OSMAN CEM YILMAZ

BURAK GÜMÜŞ

MERVE DUMAN KAVUK

MEHMET SÜHA YÜKSEL

Bu belge, 6769 sayılı Sınai Mülkiyet Kanunu kapsamında 27.12.2023 tarihinden itibaren 10 yıl süre ile korunmak üzere 23/06/2025 tarihinde verilmiştir.

Prof. Dr. M. Zeki DURAK
Başkan
